# Is there a role for Digital X-ray Radiogrammetry as surrogate marker for radiological progression and imaging of structural integrity in rheumatoid arthritis?

**DOI:** 10.1186/s12891-015-0577-3

**Published:** 2015-06-23

**Authors:** Alexander Pfeil, Peter Oelzner, Diane M. Renz, Andreas Hansch, Gunter Wolf, Joachim Böttcher

**Affiliations:** Department of Internal Medicine III, Jena University Hospital – Friedrich Schiller University Jena, Erlanger Allee 101, 07747 Jena, Germany; Institute of Diagnostic and Interventional Radiology, Jena University Hospital – Friedrich Schiller University Jena, Erlanger Allee 101, 07747 Jena, Germany; Institute of Diagnostic and Interventional Radiology, Heinrich – Braun – Clinic Zwickau, Karl-Kreil-Straße 35, 08060 Zwickau, Germany; Institute of Diagnostic and Interventional Radiology, SRH Wald-Klinikum Gera GmbH, Straße des Friedens 122, 07548 Gera, Germany

**Keywords:** Sharp score, Digital X-ray radiogrammetry, Rheumatoid arthritis, Bone mineral density, Structural integrity, Radiological progression

## Abstract

**Introduction:**

The established scoring techniques based on radiographs present limitations in the evaluation of structural integrity due to high effectiveness of innovative therapeutic strategies. The aim of this study was to evaluate the periarticular mineralisation as detected by Digital X-ray Radiogrammetry (DXR) as surrogate marker for structural integrity during the course of rheumatoid arthritis (RA).

**Methods:**

11 centers throughout Germany contributed data of 94 patients with verified RA. The patients were treated with leflunomide or methotrexate during a mean observation period of 22 months. All patients underwent complete computerized calculations of bone mineral density (BMD) and metacarpal index (MCI) by DXR using digitized hand radiographs. The radiological assessment of disease progression was estimated by the Sharp Score.

**Results:**

The Sharp Score revealed no significant change during the study period. DXR-BMD revealed minimal decrease of −1.4 % (leflunomide group) versus a higher reduction of −4.3 % (methotrexate group). Regarding DXR-MCI, a reduction of −2.2 % (leflunomide group) and −4.9 % (methotrexate group) was observed.

**Conclusion:**

Quantitative data of hand bone mass estimated by the presented DXR-technique may be a complementary precise tool in the identification of RA-related radiographic changes and in the assessment of structural integrity.

## Background

Rheumatoid arthritis (RA) is a systemic inflammatory disease characterized by progressive joint destruction and relevant disability based on the synovitis of large articulations and in particular of the peripheral joints of the hand as well as feet [1, 2].

Plain radiography has been employed as the gold standard diagnostic tool for evaluating disease progression and effectiveness of therapy in RA for many years, both in individual patients and clinical trials. Numerous scoring methods have been proposed and validated to reliably quantify radiographic visible joint damage during the course of RA [3–6].

Periarticular osteoporosis and joint space alterations are the first RA-related morphological signs before bone erosions occur [7–9]. Quantitative hand bone measurements by Digital X-ray Radiogrammetry (DXR), which capture periarticular osteoporosis [10], have been proposed as outcome measures in RA in cross-sectional studies and also as surrogate marker for radiological disease progression [9, 11–16].

The introduction of effective therapeutic strategies in the treatment of RA focused on the visualization of structural integrity rather than the inhibition of radiographic progression [17]. Acutally, the established scoring techniques present upcoming limitations in the evaluation of structural integrity based on the high effectiveness of therapeutic strategies, the recent beginning of study inclusion before erosions occur and the limited disease-related morphological differences between the study groups.

The objective of this prospective longitudinal multicenter study was the quantification of periarticular demineralization by DXR in comparison to the established Sharp Score in the evaluation of radiological progression and structural integrity.

## Methods

### Patient

In this prospectively planned, comparative, multicenter study with retrospective data analysis, patients suffering from RA were included between February and October 2010 by German rheumatologists based on the LEMERADIX-Register. All patients had to fulfill the following criteria before study inclusion:

- Confirmed diagnosis of RA according to the American College of Rheumatology criteria of 1988 [18] with different disease activities.
- Monotherapy with either leflunomide (LEF) or methotrexate (MTX) during the entire documentation period.
- Treatment with LEF in the case of contraindication of MTX (e. g. gastrointestinal ulcers, active hepatic diseases, bone marrow suppression) concerning European League against rheumatism recommendations for the treatment of RA [19].
- No combination therapy of LEF or MTX with other disease modifying anti-rheumatic drugs (DMARD) or biologicals.
- The treatment with oral glucocorticosteroids was allowed for both study cohorts.
- No intake of bisphosphonates or hormone replacement therapy during the documentation period.
- Prior use of LEF or MTX.
- Available X-ray of one hand at start of therapy with LEF or MTX (±3 months).
- Available X-ray of the same hand from the time period 1 to 3 years after start of therapy with LEF or MTX.
- Age ≥ 18 years.
- Patient informed consent prior to inclusion.

#### Acquisition of hand radiographs

All plain radiographs in anterior-posterior projection were acquired using digital X-ray equipments.

### Sharp-score

The severity of RA was evaluated using the Sharp scores with the joint space narrowing as well as the erosion score segment [20] which evaluates joints of the hands by two independent readers (blinded to each other) as follows:

- Sharp Erosion Score which evaluates 34 joints of the hands (total sum of points: 170).
- Sharp Joint Space Narrowing Score, which evaluates 36 joints of the hands (total sum of points: 144).

The individual sum of scoring points was then divided by the number of evaluated joints. If there was ambiguity in the blinded assessment, a third radiologist reviewed the radiographs and provided the final decision. The readers of the radiographs were blinded to the treatment groups.

#### Measurement of metacarpal bone mass (by digital X-ray radiogrammetry)

Digital X-ray radiogrammetry (Pronosco X-Posure System™, Version 2.0; Sectra; Sweden) was applied to determine the bone mineral density (BMD in g/cm^2^), cortical thickness (CT in cm), metacarpal bone width (W in cm) and metacarpal index (MCI; a dimensionless parameter based on the mean cortical thickness normalized with the mean outer bone diameter of the metacarpals), requiring radiographs of the non-dominant hand [21]. The radiographs were subsequently scanned into the system at a resolution of 300 dots per inch, corresponding to 5.9 line pairs/mm. The computer algorithms automatically defined regions of interest around the narrowest bone parts (i. e. diaphysis) of the metacarpals II, III and IV and subsequently determined the outer and inner cortical edges of the cortical metacarpal bone parts. The DXR-technique automatically estimates DXR-BMD in g/cm^2^, DXR-MCI (a dimensionless parameter), DXR-CT in cm and DXR-W in cm [22]. Regarding detailed technical information see also Pfeil A et al. 2011 [16].

### Ethics

The study is an non-interventional study with an retrospective analysis of pre-existing data in different centers in Germany. The study protocol was approved by the Ethics Committee of the Friedrich-Schiller-University Jena (number 2714-12/09) for all participating units. All patients received oral and written information prior to inclusion, and consented to participate by signing the informed-consent document. On a special note, the authors emphasize that all radiographs used for DXR calculations were performed as part of routine clinical care. No additional radiographs were obtained only for study purposes.

### Statistical analysis

The primary objective of the statistical analysis was to quantify the changes of the Sharp Sore and the DXR-parameters in patients with rheumatoid arthritis under therapy with MTX and LEF. The changes from baseline to month 22 were considered, X-ray imaging were compared within the groups by Wilcoxon signed-rank tests using a significance level of p < 0.05 respectively. The statistical analysis was performed using SPSS® version 15.0 (SPSS, Chicago, Illinois, USA), for Windows.

## Results

### Baseline data

A total of 11 centers throughout Germany contributed data of 94 patients. The detailed clinical patient characteristics are given in Table 1. The mean time from RA symptoms to diagnosis was 56 ± 90 months and the time from the diagnosis to the inclusion in the study was 29 ± 67 months. The mean observation period was 22 ± 8 months. Of the 94 patients included in the efficacy analysis set, 53 were treated on average with 15 ± 3.5 mg MTX and 41 patients achieved LEF (10 mg: 5 patients, 20 mg: 36 patients).

**Table 1:** Characterization of the study cohort

	Methotrexate group	Leflunomide group	Total study group
Total	n = 53	n = 41	n = 94
Women	n = 37	n = 31	n = 68
Men	n = 16	n = 10	n = 26
Age (years; mean ± SD)	55.7 ± 13.6	53.6 ± 12.4	54.8 ± 13.0
Rheumatoid factor positive	66 %	61 %	64 %
ACPA positive	58 %	51 %	55 %
CRP (mg/L, mean ± SD)	14.48 ± 24.25	15.81 ± 20.32	15.06 ± 22.00
ESR (mm/hour, mean ± SD)	28.57 ± 25.52	26.34 ± 18.67	27.60 ± 22.69
DAS28 (mean ± SD)	4.5 ± 1.1	4.2 ± 1.1	4.3 ± 1.1
DMARD naive	89 %	66 %	79 %
Corticosteroids	79 % (n = 42)	73 % (n = 30)	77 % (n = 72)
<5 mg	n = 17	n = 15	n = 32
5-10 mg	n = 25	n = 15	n = 40
Sharp Joint Space Narrowing Score (median ± SD)	1.0 ± 0.9	1.0 ± 0.8	1.0 ± 0.9
Sharp Erosion Score (median ± SD)	2.0 ± 1.0	2.0 ± 1.2	2.0 ± 1.1

Notes: ACPA = Antibodies to citrullinated proteins

CRP = C-reactive Protein

DAS = Disease Activity Score

DMARD = Disease-Modifying Anti-Rheumatic Drug

ESR = Erythrocyten Sedimentations Rate

SD = Standard Deviation

### Influence of inflammatory activity

#### Erythrocyte Sedimentation Rate (ESR) and C- reactive protein (CRP)

The ESR showed a mean decrease ± SD of 5.59 ± 13.70 mm (from 26.08 mm to 20.49 mm) in the LEF group and a mean decrease of 8.46 ± 18.25 mm (from 28.02 mm to 19.57 mm) in the MTX. The CRP showed a mean decline ± SD of 8.04 ± 23.65 mg/l (from 15.81 mg/l to 7.77 mg/l) in the LEF group and a mean decline of 5.64 ± 18.74 mg/l (from 14.48 mg/l to 8.84 mg/l) in the MTX group.

#### Disease Activity Score (DAS28

The LEF group presented an change of the DAS28 of −1.2 ± 1.4 (from 4.2 to 3.0). Regarding the MTX group a mean decline of −1.1 ± 1.1(from 4.5 to 3.4) was observed.

#### Radiological progression

For both treatment groups (LEF versus MTX) no significant changes of the Sharp Erosion Score and Sharp Joint Space Narrowing Score were observed. The median Sharp Erosion Score and Sharp Joint Space Narrowing Score was 1 at baseline. At follow up (month 22) the median Sharp Erosion Score and Sharp Joint Space Narrowing Score was also 1.

#### Methotrexate group (see Table 2 and Figure 1)

**Table 2:** Changes of DXR-parameters between baseline and month 22 for the leflunomide and methotrexate group

Group		Baseline	Month 22	Difference	Relative change	p-value
		mean (SD)	mean (SD)	mean (SD)		
Leflunomide	DXR-BMD in g/cm^2^	0.569 (0.088)	0.561 (0.094)	−0.008 (0.024)	- 1.4 %	p < 0.05
	DXR-MCI	0.452 (0.095)	0.442 (0.098)	−0.010 (0.022)	- 2.2 %	p < 0.05
	DXR-CT in cm	0.185 (0.038)	0.181 (0.040)	−0.004 (0.009)	- 2.2 %	p < 0.05
	DXR-Win cm	0.822 (0.073)	0.823 (0.073)	0.001 (0.006)	+0.1 %	p = n. s.
Methotrexate	DXR-BMD in g/cm^2^	0.578 (0.070)	0.553 (0.080)	−0.024 (0.037)	- 4.3 %	p < 0.05
	DXR-MCI	0.447 (0.076)	0.425 (0.078)	−0.022 (0.027)	- 4.9 %	p < 0.05
	DXR-CT in cm	0.186 (0.029)	0.176 (0.032)	−0.010 (0.014)	- 5.4 %	p < 0.05
	DXR-in cm	0.840 (0.086	0.835 (0.093)	−0.005 (0.031)	- 0.6 %	p = n. s.

Notes: DXR = Digital X-ray Radiogrammetry

BMD = Bone Mineral Density

MCI = Metacarpal Index

CT = Cortical Thickness

W = Metacarpal Bone Width

SD = Standard Deviation

n. s. = not significant

**Fig. 1:**
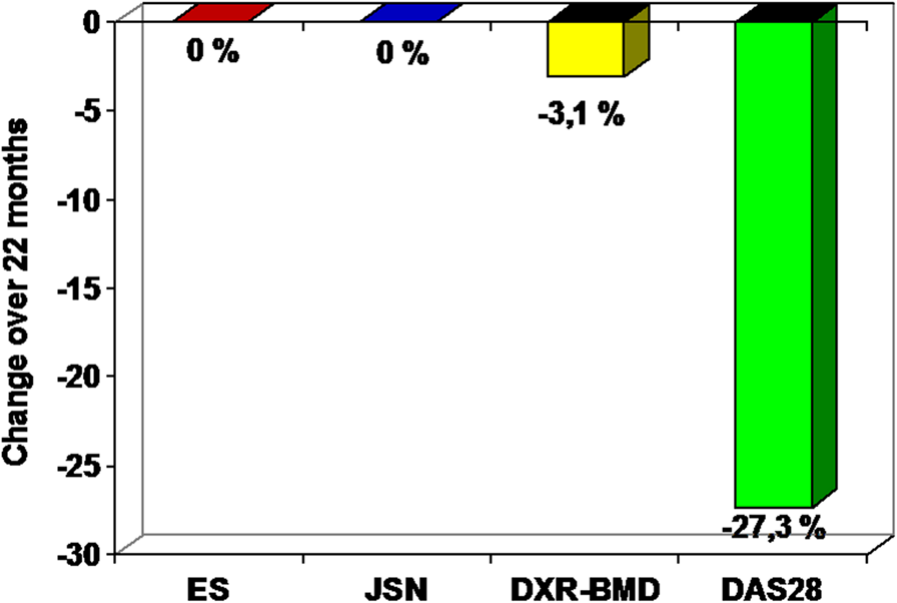
The change of the Sharp Score (ES = Sharp Erosion Score, JSN = Sharp Joint Space Narrowing Score), Bone Mineral Density estimated by DXR (DXR-BMD) and the Disease Activity Score 28 (DAS 28) for the stdy cohort (n = 94) during the observation period of 22 months

DXR-BMD in the MTX group was significantly reduced (-4.3 %) from 0.578 g/cm^2^ ± 0.070 g/cm^2^ (baseline) to 0.553 g/cm^2^ ± 0.080 g/cm^2^ (month 22). Regarding DXR-MCI (−4.9 %) and DXR-CT (−5.4 %), a significant decrease from 0.447 ± 0.076 (baseline) to 0.425 ± 0.078 (month 22) and from 0.186 cm ± 0.029 cm (baseline) to 0.176 cm ± 0.032 cm (month 22) was revealed. DXR-W presented a mild reduction (−0.6 %) from 0.840 cm ± 0.086 cm (baseline) to 0.835 cm ± 0.093 cm (month 22).

#### Leflunomide group (see Table 2 and Figure 1)

For the 41 patients treated with LEF, DXR-BMD decreased (−1.4 %) from 0.569 g/cm^2^ ± 0.088 g/cm^2^ (baseline) to 0.561 g/cm^2^ ± 0.094 g/cm^2^ (month 22). A reduction from 0.452 ± 0.095 (baseline) to 0.442 ± 0.098 (month 22) was observed for DXR-MCI (-2.2 %) and also from 0.185 cm ± 0.038 cm (baseline) to 0.181 cm ± 0.040 cm (month 22) for DXR-CT (−2.2 %). Additionally, DXR-W increased (+0.1 %) from 0.822 cm ± 0.073 cm (baseline) to 0.823 cm ± 0.073 cm (month 22).

## Discussion

It is well known that irreversible joint damage in RA occurs soon after the onset of symptoms, often within the first two years [13]. Early and effective targeted treatment, for instance with DMARDs, is required to prevent joint destruction.

Among the different DMARDs currently used in the treatment of RA, MTX is most frequently prescribed. Several studies also confirmed the efficacy of LEF on inflammation control and on radiological progression in RA [23–25].

Actually this trial is the first longitudinal study which compares the established Sharp Score and the DXR-technique focusing on the structural integrity. Our study revealed a significantly more pronounced reduction of DXR-BMD (−3.1 %) for the total study cohort during an observation period of 22 months. No difference in the effectiveness of therapy could be illustrated using the Sharp Score. Additionally, the study presented no longitudinal change of the Sharp Score for both treatment groups during the observation period. The limitation of the established scoring techniques concerning the detection of structural integrity is also related to the fact that radiological progression based on the use of effective DMARDs as control group resulting in a low radiological progression rate which is not visible by a Scoring technique. On the other hand a misinterpretation of effectiveness comparing different therapeutic strategies, in particular consideration of patients with non-erosive RA, exists.

These results are confirmed by this study showing differences between both treatment groups using DXR, but no differences could be documented by detailed scoring. However, DXR-BMD can be recommended as outcome measure and seems to be a valid surrogate marker for structural integrity caused by the similar pathogenetic mechanism as radiographic bone damage, because periarticular demineralization is still present, even if radiographic visible joint damage on X-rays apparently is arrested [26]. Altogether, these recent data showed that the DXR-technique as a precise and reliable tool could distinguish the effectiveness of MTX versus LEF therapy. Otherwise, potential limitation of this study was the absence of randomisation. The treatment of bisphosphonates and hormone replacement therapy was not allowed based on the bone protective effects of these treatment regimes which can potentially influence the result of DXR-measurements [27]. In this context, more objective data about the measurement of structural integrity in RA are now available due to computer based techniques like DXR [27], considering minor differences of structural integrity in the assessment of therapeutic strategies. Finally, the computer based quantification of radiological progression can be improved by the combined analysis of periarticular mineralization, joint space widths and identification of erosions by only one computer system in one step.

## Conclusion

Quantitative data of hand bone mass estimated by the presented DXR-technique may be a complementary precise tool in the identification of RA-related stigmata and their changes due to therapeutic strategies. The modern, more effective and early treatment of RA is associated with limited differences between treatment groups elucidating a reduced impact of scoring methods in the assessment of radiological progression. DXR could close this gap considering also small differences of structural integrity.

## Abbreviations

ACPAs: Antibodies to citrullinated proteins
CRP: C-reactive Protein
CV: Coefficient of Variation
DAS: Disease Activity Score
DMARD: Disease-Modifying Anti-Rheumatic Drug
DXR: Digital X-ray Radiogrammetry
DXR-BMD: Bone Mineral Density (g/cm^2^) estimated by Digital X-ray Radiogrammetry
DXR-MCI: Metacarpal Index estimated by Digital X-ray Radiogrammetry
ESR: Erythrocyte Sedimentation Rate
LEF: Leflunomide
MTX: Methotrexate
n. s: not significant
RA: Rheumatoid Arthritis
SD: Standard Deviation
